# Immunohistochemical detection of procalcitonin in fibrolamellar hepatocellular carcinoma

**DOI:** 10.1007/s12328-021-01354-1

**Published:** 2021-02-10

**Authors:** Kotaro Matsumoto, Kentaro Kikuchi, Ayako Hara, Hiromichi Tsunashima, Koichi Tsuneyama, Shinpei Doi

**Affiliations:** 1 Department of Gastroenterology, Teikyo University Mizonokuchi Hospital, Kawasaki, Japan; 2 Fourth Department of Internal Medicine, Teikyo University Mizonokuchi Hospital, Kawasaki, Japan; 3 Department of Pathology and Laboratory Medicine, Institute of Biomedical Sciences, Tokushima University Graduate School, Tokushima, Japan

**Keywords:** Fibrolamellar hepatocellular carcinoma, Procalcitonin, Immunohistochemical examination

## Abstract

A 25-year-old woman with fever and epigastric pain was referred to our hospital. Blood examination showed significant liver dysfunction, markedly high C-reactive protein (CRP 19.1 mg/dL) and procalcitonin (48.3 ng/mL) levels. Dynamic computed tomography showed a tumor approximately 120 mm in size in the right lobe of the liver, but with no abscess formation. The patient was hospitalized and started on antibiotics; her CRP level improved, but the procalcitonin level did not decrease. Histopathological examination of the liver tumor biopsy revealed fibrolamellar hepatocellular carcinoma (FLC). Positive staining of the FLC with an anti-procalcitonin antibody suggested the production of procalcitonin.

## Introduction

Patients with hepatocellular carcinoma manifest a variety of paraneoplastic syndromes, such as hypercholesterolemia, hypoglycemia, hypercalcemia, and erythrocytosis. This carcinoma is associated with a larger tumor volume, a higher serum alpha-fetoprotein, and a poor prognosis [1].

Fibrolamellar hepatocellular carcinoma (FLC) is a rare form of primary liver cancer and is known to occur predominantly in young adults who typically have normal background liver functions and serum α-fetoprotein (AFP) values. It accounts for 1–2% of primary liver cancer in Europe and the United States [2], but the incidence in Japan is low [3, 4].

Here, we present a patient with FLC who had abnormally high levels of procalcitonin without obvious bacterial infection. The cancer tissue obtained by biopsy was positively stained with an anti-procalcitonin antibody; therefore, the production of procalcitonin from the FLC was suggested.

## Case report

A 25-year-old woman presented with a temperature of 38 °C and epigastric pain to a nearby clinic. She had lost approximately 3 kg in the past few months, had marked liver dysfunction, and an elevated level of C-reactive protein (CRP). On referral to our hospital, her liver was palpated in the right subcostal region, and a large liver mass was found on the right lobe on abdominal ultrasound examination. She did not smoke or drink alcohol. Furthermore, she was not being treated with medications and had never taken oral contraceptives. She had no family history of liver disease.

Upon admission, her body mass index was 19.6 kg/m^2^. Blood tests showed liver dysfunction (aspartate aminotransferase, 404 U/L; alanine aminotransferase, 418 U/L; lactate dehydrogenase, 919 U/L; alkaline phosphatase, 331 U/L; gamma-glutamyl transpeptidase, 86 U/L) and inflammatory status (white blood cells [WBC]: 11,400/µL, CRP: 19.1 mg/dL). Moreover, a markedly high procalcitonin level of 48.3 ng/mL (cut-off value: 0.05 ng/mL) was observed (Table 1). She tested negative for hepatitis B surface antigen and hepatitis C virus antibody; levels of tumor markers, alpha-fetoprotein, carcinoembryonic antigen, cancer antigen 19–9, neuron-specific enolase (NSE), and progastrin-releasing peptide (ProGRP) were within the standard values.

**Table 1:** Laboratory data on admission

*Peripheral blood*		*Blood chemistry*		*Serological tests*	
WBC	11,400/μL	TP	7.0 g/dL	Procalcitonin	48.31 ng/mL
Neutro	77.2%	Alb	3.4 g/dL	Hyaluronic acid	27.8 ng/mL
Eos	0.2%	T-Bil	1.1 mg/dL	M2BPGi	(–) 0.52
Baso	0.1%	AST	404 U/L		
Mono	9.2%	ALT	418 U/L	*Tumor markers*	
Lymph	13.3%	LDH	919 U/L	AFP	6.9 ng/mL
RBC	428 × 10^4^/μL	ALP	331 U/L	CEA	< 0.5 ng/mL
Hb	12.2 g/dL	γ-GTP	86 U/L	CA19-9	< 2.0 U/mL
Plt	36.5 × 10^4^/μL	Amyl	59 U/L	NSE	8.1 ng/mL
		ChE	251 U/L	ProGRP	17.8 pg/mL
		CK	50 U/L		
		T-Chol	144 mg/dL	*Endocrine exam*	
		TG	48 mg/dL	TSH	2.670 µIU/mL
*Blood coagulation factors*		UN	9.0 mg/dL	FT4	1.29 ng/dL
PT	85.1%	Cr	0.58 mg/dL		
APTT	39.6 s	Na	137 mEq/L	*Virus markers*	
Fib	> 800 mg/dL	K	4.0 mEq/L	HBs-Ag	(–)
FDP	6.5 µg/mL	Glu	98 mg/dL	HBc-Ab	(–)
D-dimer	1.5 µg/mL	CRP	19.12 mg/dL	HCV-Ab	(–)

*γ-GTP* γ-glutamyltransferase, *AFP* α-fetoprotein, *Alb* albumin, *ALP* alkaline phosphatase, *ALT* alanine aminotransferase, *Amyl* amylase, *APTT* activated partial thromboplastin time, *AST* aspartate aminotransferase, *Baso* basophils, *CA19-9* carbohydrate antigen 19–9, *CEA* carcinoembryonic antigen, ChE: cholinesterase, *CK* creatine kinase, *Cr* creatinine, *CRP* C-reactive protein, *Eos* eosinophils, *FDP* fibrin/fibrinogen degradation products, *Fib* fibrinogen, *FT4* free thyroxine, *Glu* glucose, *Hb* hemoglobin, *HBc-Ab* hepatitis B core antibody, *HBs-Ag* hepatitis B surface antigen, *HCV-RNA* hepatitis C virus RNA, *K* potassium, *Lymph* lymphocytes, *LDH* lactate dehydrogenase, *Mono* monocytes, *M2BPGi* Mac2-binding Protein Glucosylation Isomer, *Na* sodium, *Neutro* neutrophils, *NSE* neuron-specific enolase, *P-Amyl* pancreatic amylase, *Plt* platelet count, *ProGRP* pro-gastrin-releasing peptide, *PT* prothrombin time, RBC: red blood cell count, *T-Bil* total bilirubin, *T-Chol* total cholesterol, *TG* triglycerides, *TP* total protein, *TSH* thyroid-stimulating hormone, *UN* urea nitrogen, *WBC* white blood cell count

**Fig. 1:**
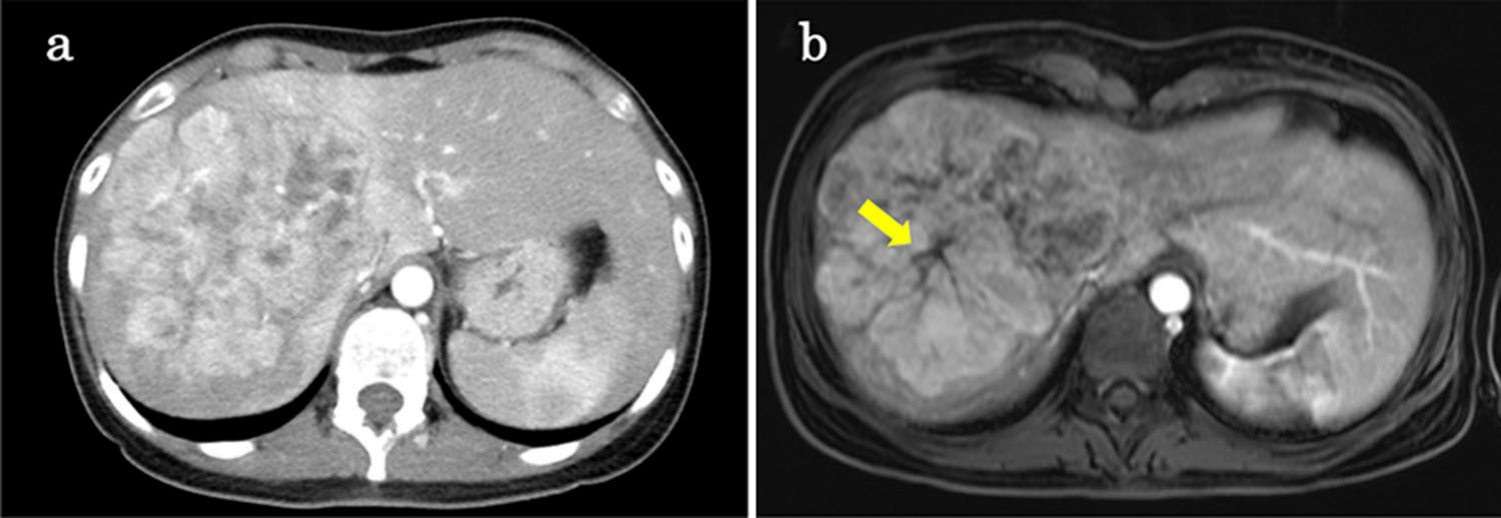
**a** Dynamic computed tomography shows a 120 mm-sized lobulated tumor occupying the entire right lobe of the liver with heterogeneous enhancement at early phase. **b** Gadolinium ethoxybenzyl diethylenetriamine pentaacetic acid-enhanced magnetic resonance imaging shows a higher signal tumor compared to the hepatic cells in the early phase, and the center of the tumor resembles scar tissue (arrows)

Dynamic computed tomography showed a 120 mm-sized lobulated tumor occupying the entire right lobe of the liver with heterogeneous enhancement at the early phase (Fig. 1a). On gadolinium ethoxybenzyl diethylenetriamine pentaacetic acid (Gd-EOB-DTPA)-enhanced magnetic resonance imaging, the tumor showed a higher signal compared to the hepatic cells in the early phase, and the center of the tumor resembled scar tissue (Fig. 1b). No notable abnormal findings were found in the upper and lower gastrointestinal endoscopy or thyroid ultrasonography.

**Fig. 2:**
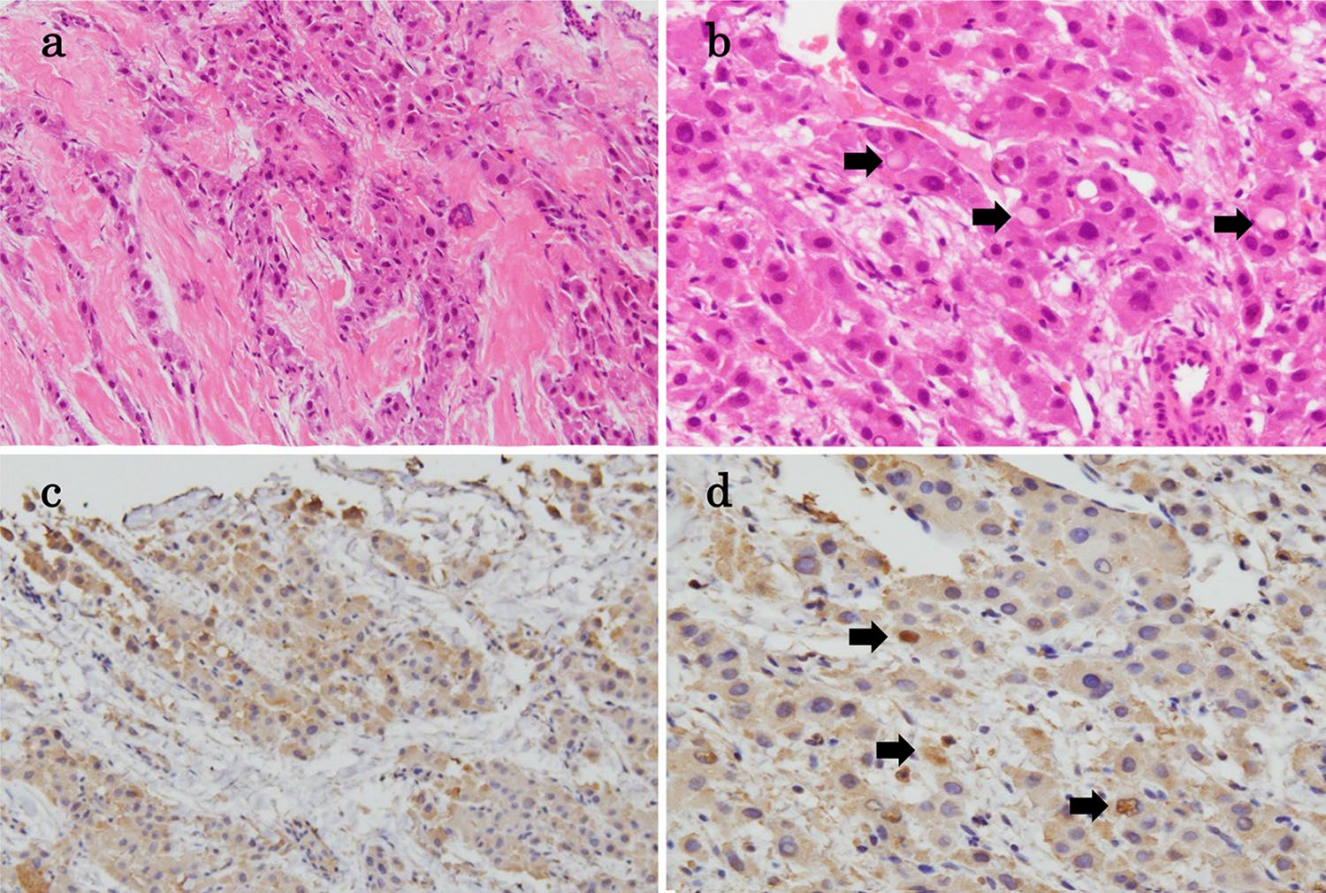
Histopathological findings of fibrolamellar hepatocellular carcinoma. **a** Tumor cells are surrounded by fibrous stroma and arranged in a lamellar distribution (Hematoxylin & Eosin staining, magnification: × 200). **b** Tumor cells showed polygonal and large with eosinophilic cytoplasm containing pale bodies (arrows) (Hematoxylin & Eosin staining, magnification: × 400). **c** The cancer tissue is positively stained by anti-procalcitonin antibody (magnification: × 200). **d** Pale bodies are also positive for anti-procalcitonin antibody (arrows) (magnification: × 400)

The patient was administered cefoperazone/sulbactam sodium after submitting two sets of blood cultures, and her WBC and CRP levels decreased. No bacteria were detected in the blood cultures, but her procalcitonin levels on the 7th and 10th hospital days were 64.6 ng/mL and 55 ng/mL, respectively. We performed an ultrasound-guided percutaneous liver tumor biopsy using an 18G-needle on the 6th day of hospitalization. The specimens were fixed in 10% buffered formalin and embedded in paraffin. Hematoxylin and eosin stains were used for the immunohistochemical staining of the following antibodies: anti-cytokeratin 7, anti-hepatocyte paraffin 1, anti-procalcitonin, anti-synaptophysin, and anti-chromogranin A. Microscopic findings showed polygonal and large tumor cells with eosinophilic cytoplasm containing pale bodies, surrounded by fibrous stroma and arranged in a lamellar distribution (Fig. 2a,b). The tumor cells were immunohistochemically positive for anti-cytokeratin 7 and anti-hepatocyte paraffin 1 antibodies. Consequently, the tumor was diagnosed as an FLC. The cancer tissue was positively stained with an anti-procalcitonin antibody (Fig. 2c, d), suggesting the production of procalcitonin from the FLC. Staining results with anti-synaptophysin antibody and anti-chromogranin A antibody were negative.

The patient was discharged from our hospital on the 11th hospital day, and she requested medical treatment at another hospital.

## Discussion

Procalcitonin is a precursor of calcitonin composed of 116 amino acids and is a biomarker for assisting diagnosis of bacterial sepsis [5]. Procalcitonin is produced when inflammatory cytokines, such as IL-6 and TNF-α, stimulate procalcitonin secretion from multiple tissue sites, such as lung, kidney, and liver [6]. The blood concentration of procalcitonin rises about 2–3 h after the onset of infection and the half-life of procalcitonin is approximately 20–24 h, making it easier to maintain high concentrations in comparison with CRP which has a half-life of 4–6 h [7, 8]. In this case, blood culture was negative, and no obvious infection focus was detected. However, cholangitis may have complicated, because the tumor occupied the entire right lobe of the liver and involved the bile duct. In addition, irregular and non-contrast-enhanced area was observed in the tumor, considering that the tumor necrosis was complicated by bacterial infection. Despite decreasing CRP after antibiotic administration, procalcitonin levels remained high for several days and the remarkable difference was observed in the change of blood concentration between procalcitonin and CRP, suggesting the possibility of procalcitonin production by malignant tumors. Thus, we performed an immunohistological examination of the liver tumor biopsy using anti-procalcitonin antibody.

High serum procalcitonin without sepsis has been reported in thyroid medullary carcinoma and some neuroendocrine cell carcinomas [9–12]. In primary liver malignancies, Han et al. reported a case of neuroendocrine carcinoma [13]. The procalcitonin level of the patient did not decline after antibiotic therapy but declined in response to transcatheter hepatic arterial chemoembolization. Meegada et al. reported a case of intrahepatic cholangiocarcinoma with high procalcitonin and paraneoplastic syndromes of hypercalcemia, polycythemia and leukocytosis, but no study have been conducted with procalcitonin antibodies [14]. In our case, the platelet count was 400,000/µL or higher during hospitalization, and 487,000/µL was observed on the 7th hospital day, suspecting reactive thrombocytosis caused by FLC.

In our case, the thyroid ultrasonography showed no obvious malignant findings. Serum levels of NSE and ProGRP were within the standard values, and immunohistological examination using anti-synaptophysin antibody and anti-chromogranin A antibody showed negative results.

In immunohistological examination, the cancer tissue was positively stained with an anti-procalcitonin antibody. Furthermore, pale bodies are also positive for anti-procalcitonin antibody. We considered that procalcitonin was produced from tumor cells of FLC and that pale body, which is plasma component, containing procalcitonin concentrated due to production from tumors or secretory disorders. On the other hand, recent studies suggest that procalcitonin level may also be increased by excessive heat stress, such as heat stroke and neuroleptic malignant syndrome [15, 16], and this case produced procalcitonin from multiple tissues in relation to tumor fever caused by FLC.

To prove this, it was important to confirm the change in procalcitonin levels after surgical treatment or chemotherapy, or procalcitonin mRNA in tumor tissue.

This case report has some limitations. First, the retrospective property of this case presentation and limited sample size could have produced possible bias. Second, this case has not been solely treated at our hospital, and the course of procalcitonin after treatment has not been followed-up. Third, genetically unproven procalcitonin production from tumor tissue.

We presented the first case of FLC with an unexpectedly high serum procalcitonin level and the cancer tissue positively stained with an anti-procalcitonin antibody. There are few reports of producing and secreting procalcitonin in primary liver malignancies including FLC. It is necessary to accumulate and examine cases in order that procalcitonin may become a useful circulating biomarker for the purpose of activity and prognosis prediction such as medullary thyroid cancer and neuroendocrine cancer previously reported [9–12].
